# Bibliometric evaluation of 2020–2022 publications on COVID-19-related cardiovascular disease

**DOI:** 10.3389/fcvm.2022.1070336

**Published:** 2023-01-13

**Authors:** Yiru Chen, Buzi Cao, Quan Zhou, Yantong Liu, Qingnan He, Mingyi Zhao

**Affiliations:** ^1^Department of Pediatrics, The Third Xiangya Hospital, Central South University, Changsha, Hunan, China; ^2^Xiangya School of Medicine, Central South University, Changsha, China; ^3^Medical School, Hunan Normal University, Changsha, China

**Keywords:** COVID-19, cardiovascular diseases, myocardial injury, heart failure, bibliometric, co-occurrence analysis, co-citation analysis

## Abstract

**Objective:**

This study aimed to investigate the international scientific output regarding the relationship between COVID-19 and cardiovascular diseases (CVDs) through a bibliometric analysis and explore research hotspots in this field.

**Methods:**

We searched the Web of Science Core Collection for publications and used different types of software, such as R, CiteSpace, and VOSviewer, to analyze and visualize the data.

**Results:**

A total of 10,055 publications were retrieved as of the 13 December 2022, based on the inclusion criteria after screening. The USA and China lead in the quantity and quality of publications in this field. Based on Bradford's law, 63 journals were considered core journals in the field. Co-cited references and keywords analysis indicated that researchers paid particular attention to cardiovascular comorbidities, outcomes, and COVID-19 regenerative medicine. In summary, with increasing COVID-19 research related to CVD, more attention might be drawn to the relationship between these two diseases.

**Conclusion:**

The hotspots in this field may continue to revolve around cardiovascular comorbidities, outcomes, and COVID-19 regenerative medicine. Owing to the different situations faced by different groups with COVID-19, further exploration of the related factors specific to each of these groups, e.g., history or no history of heart failure, is needed, with a view to providing a reference for intervention measures in COVID-19 research.

## 1. Introduction

Cardiovascular disease (CVD) is a class of diseases involving blood vessels or the heart, including coronary artery disease, cerebrovascular disease, peripheral arterial disease, rheumatic heart disease, and deep vein thrombosis. CVD is still generally considered to be the leading cause of death worldwide (1) and contributes to the decline in quality of life (2). In 2019, ischaemic heart disease accounted for 16% of the world's deaths, followed by stroke, which is responsible for ~11% of total deaths (https://www.who.int/news-room/fact-sheets/detail/the-top-10-causes-of-death).

Coronavirus disease 2019 (COVID-19) is a disease caused by severe acute respiratory syndrome coronavirus 2 (SARS-CoV-2). In addition to respiratory system dysfunction, including fever, cough, fatigue, shortness of breath, and pneumonia, which are the most common symptoms, the virus may also cause acute cardiac injury and chronic cardiovascular damage, such as myocardial injury, acute myocardial infarction, cardiac arrhythmia, and venous thromboembolism disease. Statistics have shown that COVID-19 is especially dangerous for people with pre-existing CVDs: the mortality rate of COVID-19 is approximately four times higher for patients with pre-existing CVDs (3). Additionally, the strong positive correlation between SARS-CoV-2 and CVDs was demonstrated by a study that showed that patients with cardio-cerebrovascular disease accounted for 16.4% of the 1,527 COVID-19 patients examined (4).

Possible mechanisms that underlie the relationship between CVDs and COVID-19 include systemic inflammation and ACE2-involved signaling pathways (5). Systemic inflammation occurs when the virus infects the host, triggers pattern recognition receptors, and initiates the secretion of cytokines, including interferon-α, interleukin-1, interleukin-6, and tumor necrosis factor-α (TNF-α), which is called a cytokine storm (6). For instance, as one of the most significant cytokines among all these items, TNF-α is produced by immune cells and functions by promoting epithelial cell phagocytosis and inducing endothelial cell adhesion molecules, which contribute to inflammation in nearby tissues. Meanwhile, other cytokines work in a similar way, and the aggregation of all these events is called a cytokine storm (7). On the other hand, a serine protease can catalyze the proteolytic cleavage of the S protein in SARS-CoV-2, followed by the binding of SARS-CoV-2 to ACE2. The eventual outcomes are CVDs once the conjugates have entered our cardiomyocytes (8). For the activated ACE2 signaling network, as the target of SARS-CoV-2, ACE2 not only determines the points prone to virus attack but also plays an important role in the renin-angiotensin-aldosterone system (RAAS), which greatly affects the circulatory system. In summary, an increasing number of studies are focusing on the mechanisms by which COVID-19 influences human cardiovascular health; however, to date, there is no reasonable and sufficient explanation for the series of changes that occur in the cardiovascular system after people are infected by SARS-CoV-2.

There are several methods for conducting a review, such as bibliometric analysis, systematic review, and meta-analysis. Bibliometric analysis is the use of statistical methods to analyze publications, such as articles, and it has been widely applied to obtain better knowledge about scientific research trends and identify influential publications in several areas. Compared with other categories of review, bibliometric analysis can focus on specific aspects, including authors, institutions, countries/regions, and journals, and it is relatively comprehensive. The results of bibliometric analyses have great referential value for future directions and decision making. Some previous studies have shown that bibliometric studies can provide an in-depth evaluation of research hotspots, and it is helpful for researchers to have a wide understanding of a specific research field (9–11). There have been many COVID-19-related bibliometric analyses for specific medical disciplines across the world (12–15), suggesting that bibliometrics is playing an important role in this field. We employed this method to gain insight into CVDs and COVID-19, summarize the related publications, and inspire other researchers on the way. There have already been bibliometric analyses of suicidal behavior (16), mental health (17), vaccine production (18), and other COVID-19-related research. Using bibliometric analysis, Xu et al. (19) studied cardiac involvement in COVID-19 but did not include CVDs, such as aneurysm and in-stent restenosis. This study is an unprecedented bibliometric analysis of this subject, explores the current research situation in this field, and comprehensively identifies scientific perspectives on the potential influence of COVID-19 on the human cardiovascular system and its possible contribution to immunomodulation and the increased risk of adverse outcomes.

## 2. Materials and methods

### 2.1. Data collection

The Web of Science Core Collection (WoSCC) database, including Science Citation Index Expanded (SCIE) and the Social Science Citation Index, is extensively applied in bibliometric analysis (20, 21). Data were obtained from the WoSCC as “full record and cited references” and “plain text” on a single day (13 December 2022) to prevent divergences due to daily database updates. The WoSCC was chosen because it is a curated collection of high-quality scholarly peer-reviewed literature published worldwide (22). We set up the literature search strategy based on the following keywords: “cardiomyopathy or myocardial disease or coronary artery disease or diabetic cardiomyopathies or cardiac tamponade or pericardial effusion or in stent restenosis or angina pectoris or heart failure or arrhythmia or coronary atherosclerotic heart disease or myocardial disease or heart valve sickness or pericardial disease or endocarditis or cardiac arrest or sudden cardiac death or aortic disease or aneurysm” and “COVID-19 or SARS-CoV-2.” The timespan of this bibliometric research was limited to 2020–2022, and the type of documents included were articles or reviews in English. Two reviewers (YC and BC) independently selected eligible studies for inclusion by reading the titles and abstracts. Disagreements were resolved by reaching a consensus or with the help of a third reviewer (MZ).

### 2.2. Data analysis

The WoSCC, Citespace 6.1.3R (Chaomei Chen, 2006, China), VOSviewer 1.6.18 (Nees Jan van Eck and Ludo Waltman, 2010, Holland), Microsoft Excel 2019, and the R Bibliometrix package were used to perform bibliometric analysis and visualization.

From the analysis result of the R Bibliometrix package, we easily obtained information from annual publications, which was then exported to Excel as data to forecast outputs of the next few years. Additionally, outputs of authors, countries/regions, and journals were obtained from this module (23, 24). Moreover, information about total and annual publications, total cited articles, and total times were acquired. Furthermore, the “Journal Citation Reports” of the WoSCC exhibits journal impact factor (JIF) and its JIF quartile and category.

Citespace is a bibliometric and visual analysis tool designed for observing cooperation, keywords, inner structure, potential developing trends, and dynamics in a scientific field (25, 26). Consequently, Citespace was used to visualize maps of cooperation between countries/regions and institutions, analyze co-occurring keywords, and carry out analysis of co-cited authors. Moreover, we used Citespace to carry out reference in-depth mining and cluster analysis and made the information more visible using timeline view or time zone view. The settings were listed as follows: timespan (2020–2022), years per slice (1), term source (title, abstract, author keywords, and keywords plus), pruning (none), and minimum duration of burstness (2 years); cluster labels were extracted by light semantic indexing and the log-likelihood ratio algorithm, and others followed the default (strength: cosine; scope: within slices; g-index: *k* = 25; top *N* = 50; top *N*% = 10.0%; the maximum number of selected items per slice 100; Visualization: cluster view-static and show merged network). A special case was that in the analysis of co-occurrence of references, the default software setting of *g*-index was changed to *k* = 10 and pruning-pathfinder was applied. In Citespace, the node size represents co-occurrence frequency. The nodes have various colors, which signify different years and varied from purple to yellow with time from 2020 to 2022 (25, 27, 28).

VOSviewer is also bibliometric software that is proficient at creating visualization knowledge maps, displaying them in types of clusters, overlays, and density colors (29, 30). In this bibliometric study, we used VOSviewer to draft a keyword co-occurring visualization map, in which the size of the nodes indicates the co-occurring frequency and the color of the nodes indicates the cluster. Furthermore, the link reveals the co-occurrence relationship, and the thickness of the link depends on a calculated strength value, which is proportionate to the number of publications two researchers co-authored or the number of publications in which two keywords occur together (31). In density maps, the keywords of high co-occurrence appear with larger spots. Moreover, the brighter the center of a spot is, the higher the betweenness centrality.

Microsoft Excel was used to create a trend chart of annual publications, prognosticate the number of publications in the next few years, construct a bar chart comparing the number of publications per country/region annually, generate a statistical chart of the national publications assessment index (*H*-index, citations per article, and sum of times cited), and produce tables of the information needed in this article. It was synchronously used to show information about productive countries/regions, institutions, journals, authors, and top co-cited authors and co-cited references.

## 3. Results and discussion

### 3.1. Overall

At present, an increasing number of variants of SARS-CoV-2 are emerging worldwide, the pandemic situation is still not optimistic, and the interaction between COVID-19 and CVDs has not been clearly demonstrated. More time and additional human and material resources are needed to explore and verify the exact relationship and action mechanisms between them. Therefore, it is necessary to systematically and comprehensively organize and analyze the existing global research to provide a reference for future related research.

In this study, information visualization was performed to analyze articles associated with the relationship between CVDs and COVID-19 from the Web of Science (WOS) database from 2020 to 2022. A total of 10,055 articles were included in our analysis.

**Figure 1:**
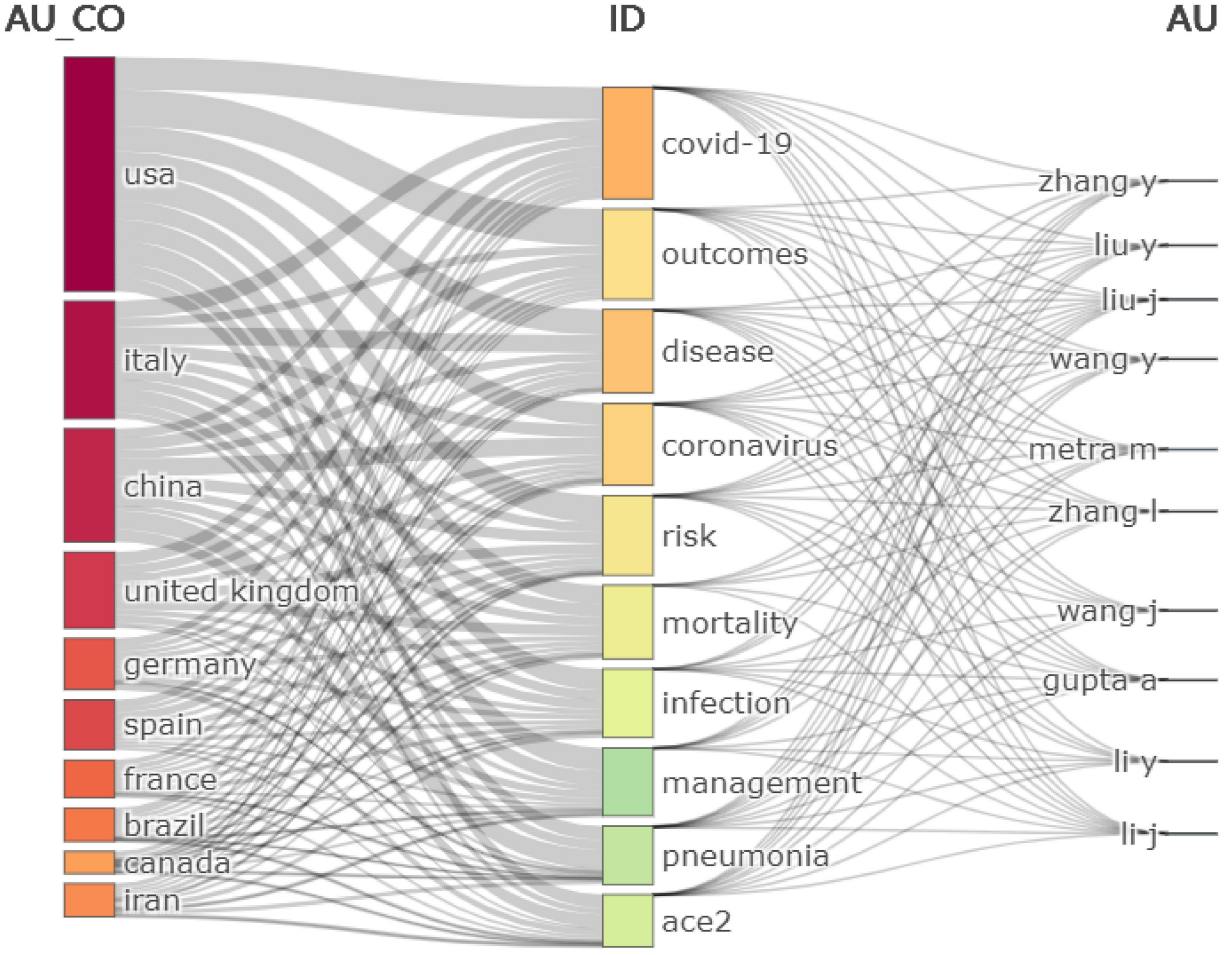
A three-field plot (Sankey diagram) of the top 10 countries with the most authors, top 10 most-cited keywords, and top 10 most productive authors.

To describe the proportion of related articles for each country and the information about citations, we created a three-field plot (Sankey diagram) of the top 10 countries with the most authors, the top 10 most productive authors, and the top 10 most cited keywords. In Figure 1, the left column shows the top 10 countries with the most authors, the middle column shows the top 10 most cited keywords, and the right column shows the top 10 most productive authors. As shown, the main interests of productive authors were COVID-19, coronavirus, and risk. In China, the main keywords were coronavirus, pneumonia, and risk. The article published by Guo et al. (32), which confirmed the significant association between myocardial injury and fatal outcomes of COVID-19, has been cited the most frequently and is considered to be one of the pioneering studies in the field of COVID-19 and CVDs. In the same year, Wichmann et al. (33) published an article in Annals of Internal Medicine and stressed the need for further studies to investigate the molecular mechanism and overall clinical incidence of COVID-19-related deaths, attracting more attention to COVID-19-induced coagulopathy. Furthermore, in the field of “COVID-19 and management,” which is one of the main interests of productive authors, Nobari and Goodarzi (34) conducted a systematic review about COVID-19 patients with specific skin disorders and management strategies, indicating that biological or immunomodulator agents should stop being used during COVID-19's acute period and should only be administered once again after treatment shows a low recurrence rate. Besides, in the Journal of the American Heart Association, Chen et al. (35) and his partners found that cardiometabolic disease is common in hospitalized COVID-19 patients, and COVID-19 patients with cardiometabolic disease have a higher risk of death than those without the disease. Moreover, Manary et al. (36) published an article in the Proceedings of the National Academy of Sciences suggesting that autoimmunity, which is related to type I IFNs, may increase the risk of the death in COVID-19 patients and IFNs are related to a patient's age.

### 3.2. Country/region and institution analysis

**Figure 2:**
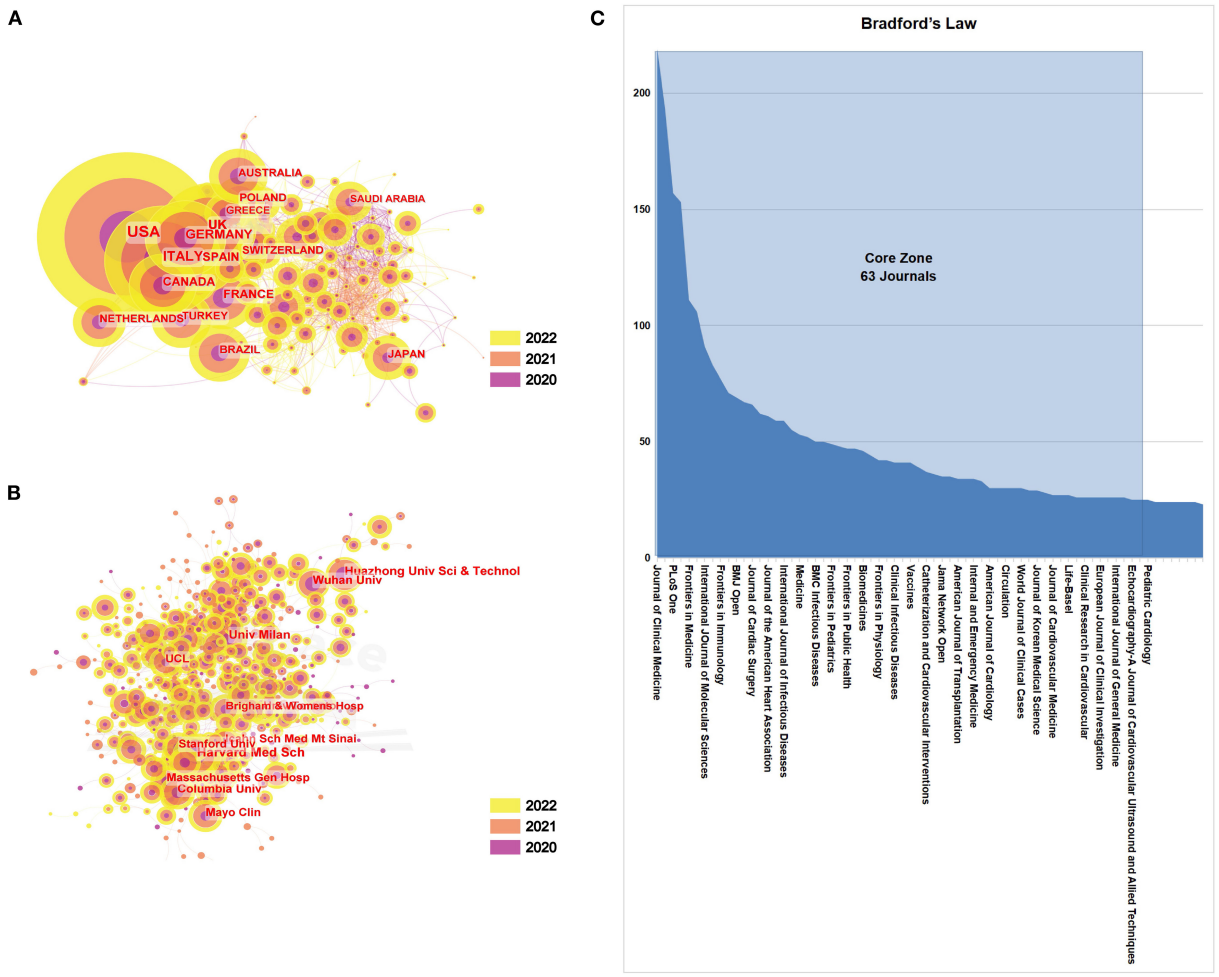
**(A)** Collaboration analysis of countries/regions involved in COVID-19-related CVD research. **(B)** Collaboration analysis of institutions involved in COVID-19-related CVD research. **(C)** Bradford's law of journals involved in COVID-19-related CVD research.

CiteSpace was used to show international and interorganizational collaboration. In Figure 2, the countries/institutions marked with purple circles feature stronger centrality, while the connection between nodes indicates collaboration between countries/institutions.

The outcome becomes slightly different for the sum of times cited and citations per article (Figure 2A, Table 1). The USA had the highest citation/article ratio (23.03%), indicating that the articles written by scholars in this country were not only numerous but also of high reference value. China had the second highest citation/article ratio (11.47%), followed by Italy (10.26%). However, it is worth noting that although France had the lowest number of articles (258) and the lowest citation/article ratio (2.57), total citations (6,535) and article citations (2,533) were comparatively high, suggesting that France had also considerably influenced this field.

**Table 1:** Top 10 most productive countries/regions for COVID-19-related CVD research.

**Rank**	**Country**	**Articles (*N*)**	**Percentage (*N*/10055)**	**Total citations**	**Average article citations**
1	USA	2,316	23.03%	68,683	29.66
2	China	1,153	11.47%	80,170	69.53
3	Italy	1,032	10.26%	21,219	20.56
4	United Kingdom	532	5.29%	19,897	37.4
5	Germany	410	4.08%	10,640	25.95
6	Turkey	347	3.45%	1,654	4.77
7	India	331	3.29%	3,995	12.07
8	Spain	276	2.74%	5,234	18.96
9	Brazil	271	2.70%	3,835	14.15
10	France	258	2.57%	6,535	25.33

Regarding institutions, the top 10 institutions are listed in Table 2. Harvard Medical School (429, 4.27%) was the leading institution devoted to this area, with the highest article percentage of all organizations. Huazhong University of Science and Technology (384, 3.82%) and Wuhan University (382, 3.80%) were second and third place, respectively, probably because they been studying this topic for a long time, as the first COVID-19 patient in the world was found in Wuhan, the city where these two universities are located. Other institutions are carrying out correlated research, including Columbia University (316, 3.14%), Tehran University of Medical Sciences (296, 2.94%), and the University of Milan (286, 2.84%). Figure 2B shows that the relationship between these institutions is increasingly active, which may be related to the academic performance and influence of the USA in this area.

**Table 2:** Top 10 most productive institutions for COVID-19-related CVD research.

**Rank**	**Institutions**	**Articles (*N*)**	**Percentage (*N*/10055)**
1	Harvard Medical School	429	4.27%
2	Huazhong University of Science and Technology	384	3.82%
3	Wuhan University	382	3.80%
4	Columbia University	316	3.14%
5	Tehran University of Medical Sciences	296	2.94%
6	University of Milan	286	2.84%
7	Icahn School of Medicine at Mount Sinai	285	2.83%
8	Mayo Clinic	258	2.57%
9	University of Paris	231	2.30%
10	University of Toronto	217	2.16%

In addition, Figures 2A, B reveals some essential information about countries/regions and institutions in the field of COVID-19 and CVDs. Figure 2A shows that the USA has made the greatest contribution in this field. Additionally, Australia, Saudi Arabia, and Japan have contributed a great deal but their research connections with other countries were relatively few. Figure 2B shows that there are close connections between institutions in the same countries/regions, such as Huazhong University of Science and Technology and Wuhan University, while institutions from different countries/regions are relatively independent, such as the University of Milan and Wuhan University.

### 3.3. Journal analysis

In total, there were 10,055 publications from 341 journals. According to Bradford's law, 63 journals were recognized as core zone in the field (Figure 2C). There was no large gap in the number of articles in the top 10 most productive journals. Table 3 presents the top 10 most productive journals in the field of CVDs. Overall, 1,261 documents were published in the top 10 most active journals, which accounted for 12.54% of all publications related to CVDs and the COVID-19 pandemic. The Journal of Clinical Medicine, which had an IF of 4.964 in 2021, published the most articles (219, 2.18%), followed by Frontiers in Cardiovascular Medicine (193, 1.92%), PLoS One (157, 1.56%), and the International Journal of Environmental Research and Public Health (153, 1.52%). It was noteworthy that PLoS One also had the highest H-index among the top 10 most productive journals. Frontiers in Immunology, which had the highest IF of 8.786 among the top 10 most productive journals, was ranked the ninth most productive in this field with 77 publications. With regard to quartile in category, four journals were in Q1 (the top 25% of the IF distribution) in different areas and six journals were in Q2 (25–50%), suggesting differences in terms of article quality.

**Table 3:** Top 10 most productive journals related to CVDs and COVID-19.

**Rank**	**Journal**	**Count (*N*)**	**Percentage (*N*/10055)**	**IF** **(2021)**	**JCI** **(2021)**	**H-index**	**Quartile** **in category**
1	Journal of Clinical Medicine	219	2.18%	4.964	1.02	23	Q2^a^
2	Frontiers in Cardiovascular Medicine	193	1.92%	5.846	0.91	16	Q2^b^
3	PLoS One	157	1.56%	3.752	0.88	26	Q2^c^
4	International Journal of Environmental Research and Public Health	153	1.52%	3.229	0.93	17	Q1^d^
5	Frontiers in Medicine	111	1.10%	5.058	0.97	15	Q2^a^
6	Scientific Reports	106	1.05%	4.996	1.05	16	Q2^c^
7	International Journal of Molecular Sciences	91	0.91%	6.208	0.70	16	Q1^e^
8	ESC Heart Failure	83	0.83%	3.612	0.77	15	Q2^b^
9	Frontiers in Immunology	77	0.77%	8.786	1.01	17	Q1^f^
10	American Journal of Emergency Medicine	71	0.71%	4.093	1.23	18	Q1^g^

^a^Medicine, general, and Internal.

^b^Cardiac and cardiovascular systems.

^c^Multidisciplinary sciences.

^d^Public, environmental, and occupational health.

^e^Biochemistry and molecular biology.

^f^Immunology.

^g^Emergency medicine.

Table 4 lists the top 10 most productive co-cited journals in this field. The New England Journal of Medicine was the most productive co-cited journal (16,505 articles), the IF of which was 176.079 in 2021, and the H-index was 11, followed by The Lancet (13,838 articles), the Journal of the American Medical Association (10,067 articles), Circulation (9,225 articles), and the Journal of the American College of Cardiology (6,561 articles). In Table 4, the top 10 most co-cited journals were all in Q1 in different areas, indicating high productivity and quality.

**Table 4:** Top 10 most productive co-cited journals related to CVDs and COVID-19.

**Rank**	**Sources**	**Articles (*N*)**	**IF (2021)**	**JCR (2021)**	**H-index**	**Quartile in category**
1	New England Journal of Medicine	16,505	176.079	22.47	11	Q1^a^
2	The Lancet	13,838	202.731	21.87	3	Q1^a^
3	Jama-Journal of the American Medical Association	10,067	157.335	10.46	9	Q1^a^
4	Circulation	9,225	39.918	6.31	7	Q1^b^
5	Journal of the American College of Cardiology	6,561	6.240	4.74	28	Q1^b^
6	European Heart Journal	5,871	35.855	6.82	17	Q1^b^
7	PLoS One	5,630	3.752	0.88	26	Q1^c^
8	Nature	5,575	69.504	10.86	4	Q1^c^
9	Jama Cardiology	5,538	30.154	6.04	21	Q1^b^
10	BMJ-British Medical Journal	4,158	93.333	7.45	14	Q1^a^

^a^Medicine, general, and internal.

^b^Cardiac and cardiovascular systems.

^c^Multidisciplinary sciences.

Among these journals, Frontiers in Cardiovascular Medicine contained the greatest number of relevant articles. Somasundaram Raghavan and other scholars expanded the manifestations of CVDs caused by COVID-19, including the combination between viral S-protein and ACEs, the significance of transmembrane serine protease 2 in the proteolytic cleavage of S-protein, and the role of phosphoinositide 5-kinase, cathepsin L and two-pore segment channel 2 in the transmembrane transport of viruses (37). The review by Ellison-Hughes et al. (38) drew the conclusion that the cytokine storm caused by SARS-CoV-2 can be reduced by mesenchymal stromal cells (MSCs), which indicates that MSC transplantation has the potential to treat CVDs relevant to SARS-CoV-2. The review by Breikaa et al. pointed out that the Notch pathway is one of the mechanisms of COVID-19 related to CVDs. The Notch pathway can affect the two main contributing factors of cardiovascular complications by reducing both inflammation and coagulation (39). Besides, Ercan et al. (40) found that platelets have specific changes in COVID-19-related cardiovascular patients. In summary, Frontiers in Cardiovascular Medicine contains many relevant research papers and the latest advances and will thus contribute greatly to future research and clinical practice.

In addition, the Journal of the American Heart Association contributes greatly to this research field. In this journal, many scholars focus on angiotensin-converting enzyme inhibitors (ACE inhibitors) and angiotensin receptor blockers (ARBs). Rohan Khera and his scientific research team found that although the link between ACE inhibitors and COVID-19 was true, COVID-19 hospitalization and mortality were not strongly relevant to ACE inhibitors and ARBs (41). However, according to Pan et al. (42) clinical research, ACE inhibitors and ARBs show great curative effects in COVID-19 patients with lower levels of inflammation. These results showed the significance of further in-depth research related to the relationship between ACE inhibitors, ARBs, and SARS-CoV-2-related CVDs. Other journals have also provided many valuable research results; for instance, in the Journal of Clinical Medicine, some scholars have expanded the impact of COVID-19 on the cardiovascular system (43) and paid attention to thrombosis and COVID-19 (44). Driven by these scientific research results, SARS-CoV-2-related CVDs are attracting an increasing number of scholars.

### 3.4. Author analysis

**Figure 3:**
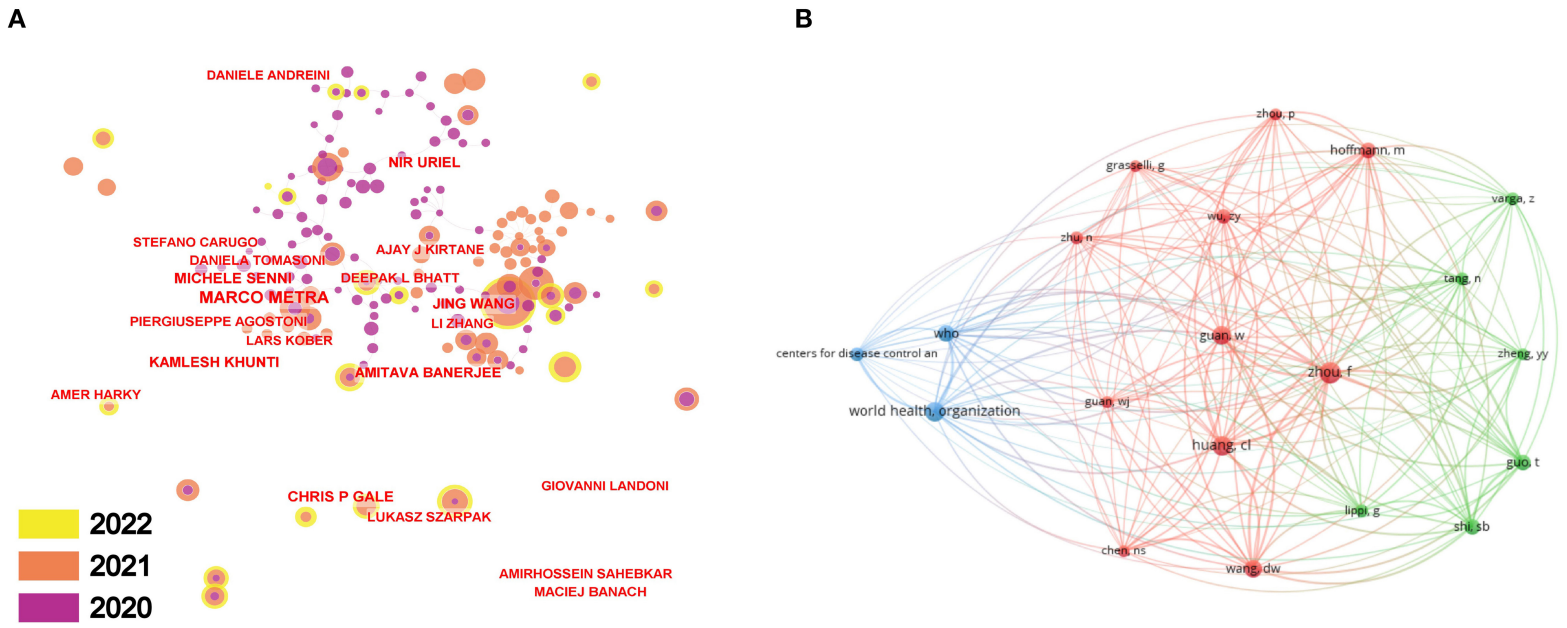
**(A)** CiteSpace network visualization map of productive authors of the articles correlated with COVID-19-related CVD research. **(B)** VOSviewer network visualization map of co-cited authors of COVID-19-related CVD research.

The top 10 most productive authors and most co-cited authors are listed in Table 5, the productive author network analysis is visualized in Figure 3A, and the co-cited author network analysis is visualized in Figure 3B. Zhang Y was the most productive author with 59 documents, focusing on traditional Chinese therapy, such as acupuncture (45) and Tai Chi used in COVID-19 patient treatment. The author summarized the potential benefits of Tai Chi for the elderly: improving cardio respiratory and microcirculatory function, lipid profile, and blood pressure (18). Next were Wang Y (45 documents), Zhang L (41 documents), Wang J (40 documents), and Liu Y (39 documents). Li H was the most locally cited author with 3,685 citations. As a radiologist, Li mainly made use of radiology techniques, such as computed tomography (CT), to evaluate disease severity in patients, and further on, to predict their overall survival (46). Li also collaborated with other scientists to explore the relationship between serum SARS-CoV-2 nucleic acid (RNAemia) and the extent of organ damage, such as cardiac damage, by providing CT as evidence, leading to the conclusion that patients with RNAemia tend to have greater abnormalities and organ damage (47).

**Table 5:** Top 10 most productive authors and co-cited authors correlated with COVID-19-related CVD research.

**Rank**	**Most relevant authors**	**Articles (*N*)**	**H-index**	**Rank**	**Most local cited authors**	**Local citations**	**H-index**
1	Zhang Y	59	17	1	Li H	3,685	11
2	Wang Y	45	15	2	Yu T	3,514	3
3	Zhang L	41	15	3	Fan GH	3,500	4
4	Wang J	40	15	4	Cao B	3,499	3
5	Liu Y	39	11	5	Gu XY	3,499	3
6	Li J	37	11	6	Xu JY	3,499	3
7	Metra M	37	17	7	Wang YW	3,493	2
8	Liu J	36	13	8	Wei Y	3,493	2
9	Gupta A	34	11	9	Zhang L	2,889	15
10	Li Y	34	12	10	Zhang Y	2,389	17

### 3.5. Co-cited references analysis

**Figure 4:**
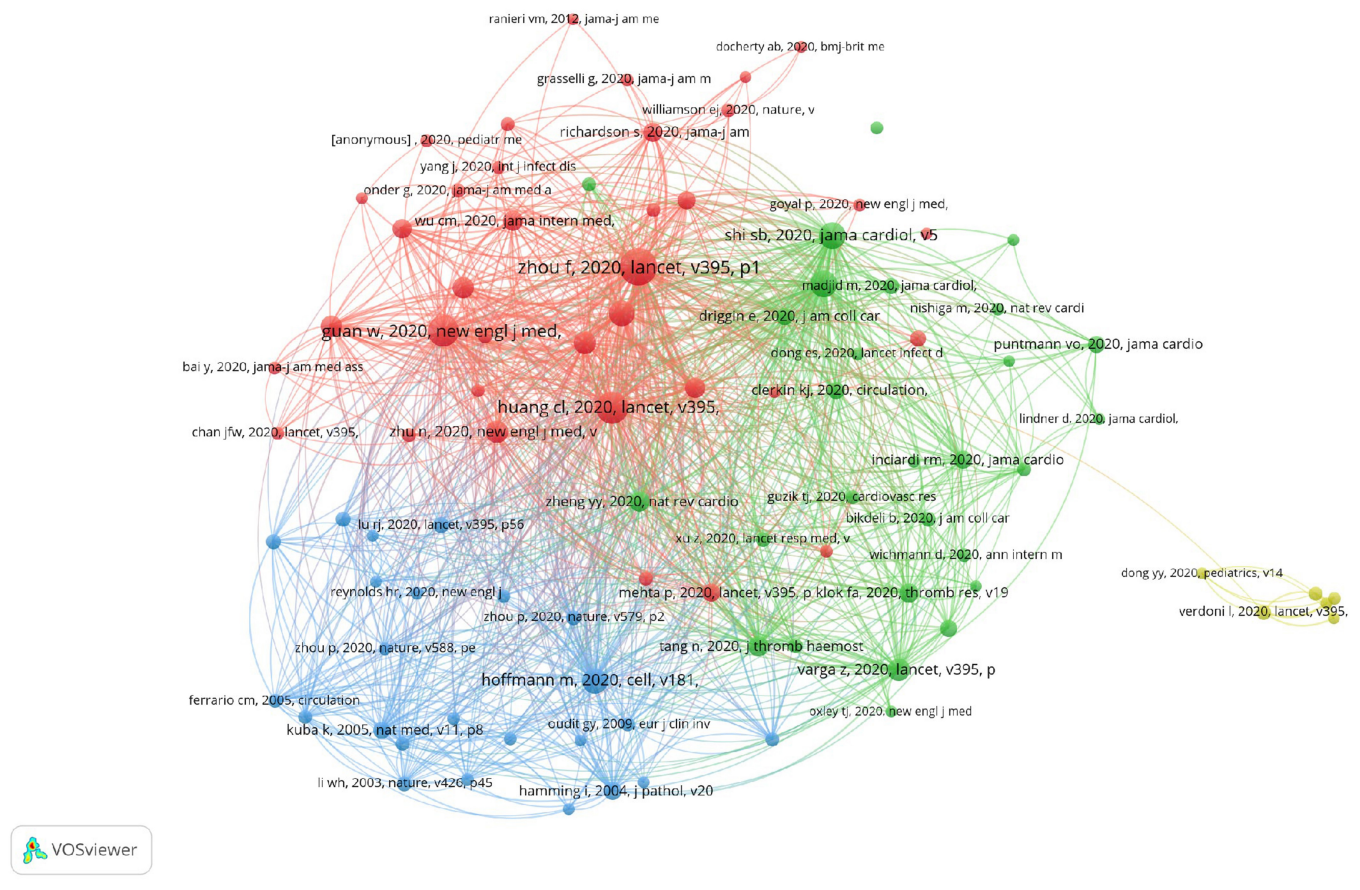
VOSviewer network visualization map of references to COVID-19-related CVD research.

Co-citation refers to the relationship between two publications when they are simultaneously referenced by one (later published) piece of literature. Figure 4 and Table 6 list the top 10 most co-cited references in research investigating CVDs in COVID-19. Most of the top co-cited authors are Chinese (8 out of 10), indicating that many devoted and diligent Chinese scientists are contributing to research on CVDs in COVID-19. It is also noticeable that their articles are published in some of the top journals, including The Lancet (48), JAMA Cardiology (49), the New England Journal of Medicine (50), and Cell (51), further indicating the quality of their work and the importance attached to this topic.

**Table 6:** Top 10 co-cited references in COVID-19-related CVD research.

**Rank**	**Title**	**Citations**	**First author**	**Journal**
1	Return-to-work, disabilities and occupational health in the age of COVID-19	1999	ZHOU F	LANCET
2	Clinical features of patients infected with 2019 novel coronavirus in Wuhan, China	1494	HUANG CL	LANCET
3	Clinical Characteristics of Coronavirus Disease 2019 in China	1412	GUAN W	NEW ENGL J MED
4	Association of Cardiac Injury with Mortality in Hospitalized Patients With COVID-19 in Wuhan, China	1059	SHI SB	JAMA CARDIOL
5	Cardiovascular Implications of Fatal Outcomes of Patients With Coronavirus Disease 2019 (COVID-19)	1053	GUO T	JAMA CARDIOL
6	Characteristics of and Important Lessons From the Coronavirus Disease 2019 (COVID-19) Outbreak in China: Summary of a Report of 72,314 Cases From the Chinese Center for Disease Control and Prevention	970	WU ZY	JAMA-J AM MED ASSOC
7	SARS-CoV-2 Cell Entry Depends on ACE2 and TMPRSS2 and is Blocked by a Clinically Proven Protease Inhibitor	954	HOFFMANN M	CELL
8	A Novel Coronavirus from Patients with Pneumonia in China, 2019	746	ZHU N	NEW ENGL J MED
9	Clinical Characteristics of 138 Hospitalized Patients With 2019 Novel Coronavirus-Infected Pneumonia in Wuhan, China	731	WANG DW	JAMA
10	Endothelial cell infection and endotheliitis in COVID-19	723	VARGA Z	LANCET

The most co-cited article, written by Zhou et al. (48) and published in The Lancet, tops the list with 1,999 citations. It was published in the early stage of the pandemic (March 2020) and systemically analyzed the clinical course and risk factors for mortality and comorbidities among COVID-19 patients, indicating its value and influence in the field of CVDs in COVID-19. The second most co-cited article was also published in The Lancet and was written by Huang et al. (52). Distinguished from the first article, Huang CL et al. recorded and analyzed the clinical features of COVID-19 patients, including comorbidities such as CVDs. It deserves its high number of citations as it clarified possible symptoms in the very early stage of the epidemic (by 2 January 2020), which is of great importance for paving the road for the development of treatments and in terms of prevention. The next article on the list was written by Guan et al. (50), published in the New England Journal of Medicine, and has been co-cited 1,412 times. This article also addressed the clinical characteristics of COVID-19 patients; however, the authors collected more data from more patients in 30 provinces and helped to further clarify the possible symptoms associated with COVID-19. Overall, the top 3 co-cited references have each been cited more than 1,400 times.

### 3.6. Keyword analysis

We exploited CiteSpace to analyze and visualize the relationship between different subjects, with the size of the nodes representing the frequency of keywords. In general, it can be concluded that CVDs in COVID-19 are associated with myocarditis, myocardial infarction, ACE2, pneumonia, etc. Zhou et al. (48) illustrated that the most common cardiovascular comorbidities among COVID-19 patients are hypertension (30%) and coronary heart disease (8%).

**Figure 5:**
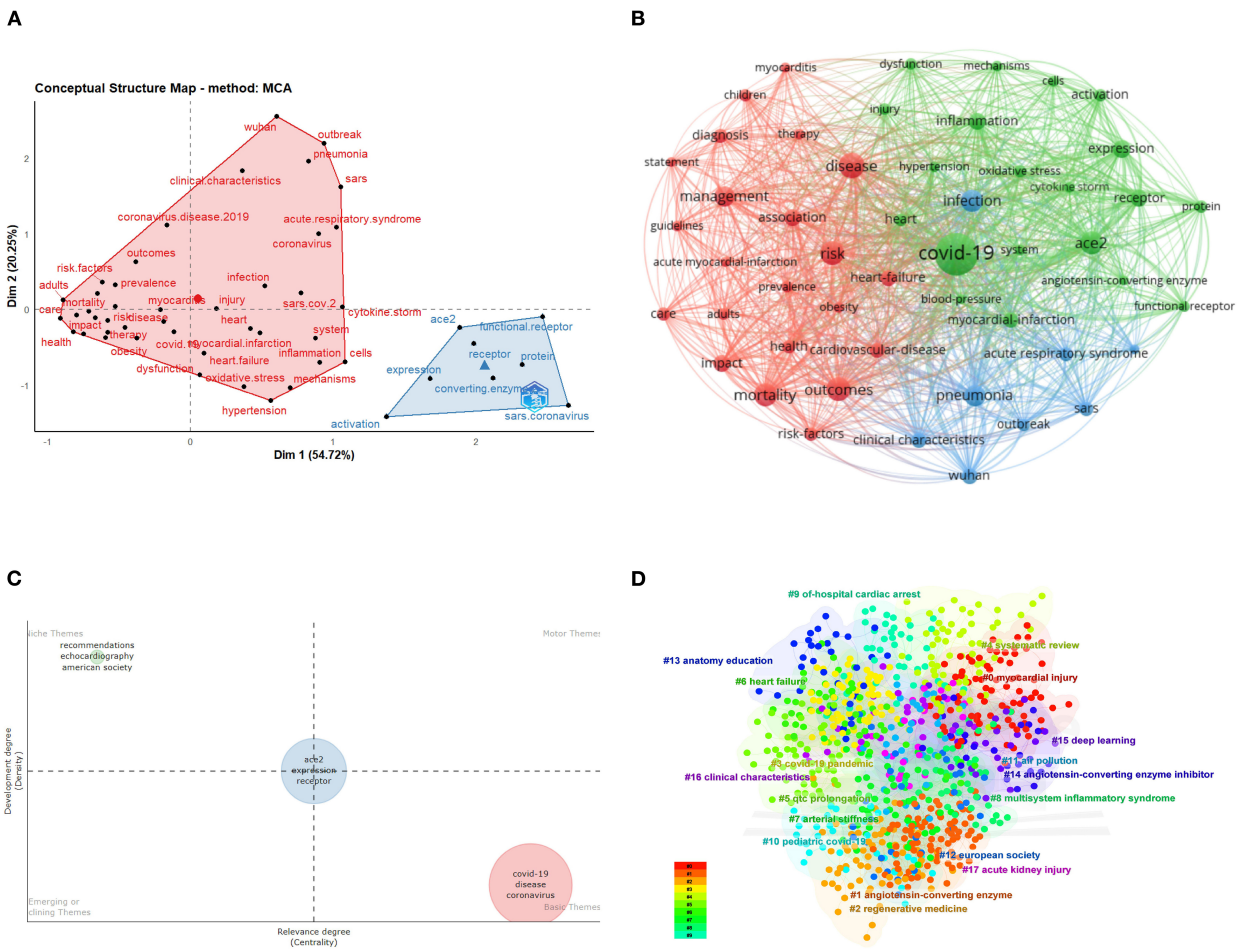
Analysis of keywords in COVID-19-related CVD research. **(A)** Factorial analysis of keywords. **(B)** Cluster analysis of keywords associated with COVID-19-related CVD research. **(C)** Thematic network map of keywords. **(D)** Citespace network visualization map of co-occurring author keywords.

In Figure 5A, multiple correspondence analysis (MCA) was performed to construct a conceptual structure of this field. All keywords were divided into two clusters in blue and red. Figure 5B shows that besides COVID-19, disease, risk, outcomes, and mortality are hotspots. Other keywords, such as heart failure and obesity, were also discussed frequently, indicating that there might be a potential correlation between COVID-19 and these CVDs. In recent studies, COVID-19 hospitalization has been reported to be associated with an increased risk of incident heart failure (53), but the specific molecular mechanism in the process remains unclear, which means the research about heart failure and COVID-19 still has much room for development. In Figure 5C, we visualized some top themes as a thematic map to better understand their characteristics. No themes were found in the upper right and lower left quadrant. The niche themes included “recommendations,” “echocardiography,” and “American society,” which means they were highly developed yet, at the same time, relatively isolated topics. “COVID-19,” “disease,” and “coronavirus” were three basic themes that were important yet underdeveloped. Moreover, “ACE2,” “expression,” and “receptor” were themes located in the boundary between motor themes and niche themes, exhibiting middle centrality and middle development degree.

In Figure 5D, we visualized a certain keyword cumulate occurrence. The largest cluster is “myocardial injury” (cluster #0), followed by “angiotensin-converting enzyme” (cluster #1), “regenerative medicine” (cluster #2), and “COVID-19 pandemic” (cluster #3). It is not surprising that myocardial injury and angiotensin-converting enzyme are hot topics in the field of CVDs in COVID-19, as myocardial injury has a relatively high incidence in COVID-19 patients (54–56) and the interaction between the spike protein receptor-binding domain of SARS-CoV-2 and angiotensin-converting enzyme 2 is a significant link in the pathogenesis of COVID-19 (31, 57). We further explored “regenerative medicine” as scholars are studying the effect of targeted protein S-nitrosylation of ACE2 (58) and medicines such as optimized ACE2 decoys (59) in the treatment of COVID-19, reflecting peoples' focus on COVID-19 therapies. As for “COVID-19 pandemic,” it was in accordance with expectations as it is universally acknowledged that the COVID-19 pandemic has brought about tremendous changes in many aspects of peoples' lives.

## 4. Conclusions

To our knowledge, this study is the first bibliometric analysis focused on the correlation between COVID-19 and CVDs. Since the outbreak of COVID-19 in 2019, an increasing number of studies have been carried out, and humans are making ongoing progress toward conquering the virus. In 2020–2022, the USA made the largest contribution to this field, and Harvard Medical School was the most productive institution. The most productive journal was the Journal of Clinical Medicine, and the most productive author was Zhang Y. The research hotspots in this field continue to revolve around cardiovascular comorbidities, outcomes, and regenerative medicine for COVID-19.

## 5. Limitations

This study had some limitations. First, although Citespace and VOSviewer can show different visual effects and various and multilevel information through multiple types of charts with the same data, the consistency of the analysis result of the two tools cannot be guaranteed. Second, non-English articles are excluded in this study, which may contribute to the source bias. Additionally, we did not conduct a comprehensive analysis of the information obtained but selectively analyzed the characteristics of these data, thus, some essential details may be overlooked. Finally, compared with other methods for conducting reviews, such as meta-analysis and systematic review, bibliometric analysis is not able to evaluate the specific contents of research results, and this needs to be further developed.

## Data availability statement

The datasets presented in this study can be found in online repositories. The names of the repository/repositories and accession number(s) can be found in the article/supplementary material.

## Author contributions

YC and MZ conceptualized and designed the study. YC and BC collected data, performed data analysis, and produced tables and figures. YC, BC, and QZ participated in writing the manuscript. YC, BC, QZ, and MZ revised the manuscript. All authors approved the final manuscript and agreed to be responsible for all aspects of the study.

## References

[1] Joseph P Leong D McKee M Anand SS Schwalm JD Teo K. Reducing the global burden of cardiovascular disease, part 1: the epidemiology and risk factors. Circ Res. (2017) 121:677–94. 10.1161/CIRCRESAHA.117.308903 28860318

[2] Mensah GA Roth GA Fuster V. The global burden of cardiovascular diseases and risk factors: 2020 and beyond. J Am Coll Cardiol. (2019) 74:2529–32. 10.1016/j.jacc.2019.10.009 31727292

[3] Dou Q Wei X Zhou K Yang S Jia P. Cardiovascular manifestations and mechanisms in patients with COVID-19. Trends Endocrinol Metab. (2020) 31:893–904. 10.1016/j.tem.2020.10.001 33172748 PMC7566786

[4] Aghaei Moghadam E Mohammadzadeh S Sattarzadeh Badkoubeh R Ghamari A Rabbani A Mohebbi A. COVID-19: a new horizon in congenital heart diseases. Front Pediatr. (2021) 9:582043. 10.3389/fped.2021.582043 34956968 PMC8693804

[5] Zheng YY Ma YT Zhang JY Xie X. COVID-19 and the cardiovascular system. Nat Rev Cardiol. (2020) 17:259–60. 10.1038/s41569-020-0360-5 32139904 PMC7095524

[6] Vabret N Britton GJ Gruber C Hegde S Kim J Kuksin M. Immunology of COVID-19: current state of the science. Immunity. (2020) 52:910–41. 10.1016/j.immuni.2020.05.002 32505227 PMC7200337

[7] Goswami SK Ranjan P Dutta RK Verma SK. Management of inflammation in cardiovascular diseases. Pharmacol Res. (2021) 173:105912. 10.1016/j.phrs.2021.105912 34562603 PMC8541927

[8] Guzik TJ Mohiddin SA Dimarco A Patel V Savvatis K Marelli-Berg FM. COVID-19 and the cardiovascular system: implications for risk assessment, diagnosis, and treatment options. Cardiovasc Res. (2020) 116:1666–87. 10.1093/cvr/cvaa106 32352535 PMC7197627

[9] You Y Min L Tang M Chen Y Ma X. Bibliometric evaluation of global tai chi research from 1980 to 2020. Int J Environ Res Public Health. (2021) 18:6150. 10.3390/ijerph18116150 34200236 PMC8201343

[10] You Y Wang D Liu J Chen Y Ma X Li W. Physical exercise in the context of air pollution: an emerging research topic. Front Physiol. (2022) 13:784705. 10.3389/fphys.2022.784705 35295574 PMC8918627

[11] You Y Li W Liu J Li X Fu Y Ma X. Bibliometric review to explore emerging high-intensity interval training in health promotion: a new century picture. Front Public Health. (2021) 9:697633. 10.3389/fpubh.2021.697633 34368063 PMC8342813

[12] Zhang Q Li J Weng L. A bibliometric analysis of COVID-19 publications in neurology by using the visual mapping method. Front Public Health. (2022) 10:937008. 10.3389/fpubh.2022.937008 35958855 PMC9362596

[13] Thornley PE Aitken JM Nichol GM Slevin NJ. Amoxycillin-clavulanic acid combination in bronchopulmonary infection due to beta-lactamase-producing branhamella catarrhalis. Prelim Rep Drugs. (1986) 31(Suppl 3):113–4. 3525087 10.2165/00003495-198600313-00024

[14] Zhang Q Li S Liu J Chen J. Global trends in nursing-related research on COVID-19: a bibliometric analysis. Front Public Health. (2022) 10:933555. 10.3389/fpubh.2022.933555 35923953 PMC9339968

[15] Soytas M Danacioglu YO Boz MY Horuz R Albayrak S. COVID-19 and urology: a bibliometric analysis of the literature. Int J Clin Pract. (2021) 75:e14965. 10.1111/ijcp.14965 34626151 PMC8646722

[16] Grover S Gupta BM Mamdapur GM. COVID-19 and suicidal behavior: a bibliometric assessment. Asian J Psychiatr. (2021) 65:102817. 10.1016/j.ajp.2021.102817 34479024 PMC8545807

[17] Chen Y Zhang X Chen S Zhang Y Wang Y Lu Q. Bibliometric analysis of mental health during the COVID-19 pandemic. Asian J Psychiatr. (2021) 65:102846. 10.1016/j.ajp.2021.102846 34562753 PMC8435062

[18] Zhu X Luo Z Chen Y Wang L Chi W Jiang LL. Tai chi for the elderly patients with COVID-19 in recovery period: a protocol for systematic review and meta-analysis. Medicine. (2021) 100:e24111. 10.1097/MD.0000000000024111 33546018 PMC7837887

[19] Xu SC Zhao XY Xing HP Wu W Zhang SY. Cardiac involvement in COVID-19: a global bibliometric and visualized analysis. Front Cardiovasc Med. (2022) 9:955237. 10.3389/fcvm.2022.955237 35966543 PMC9365052

[20] Yan WT Lu S Yang YD Ning WY Cai Y Hu XM. Research trends, hot spots and prospects for necroptosis in the field of neuroscience. Neural Regen Res. (2021) 16:1628–37. 10.4103/1673-5374.303032 33433494 PMC8323674

[21] Zhang J Song L Xu L Fan Y Wang T Tian W. Knowledge domain and emerging trends in ferroptosis research: a bibliometric and knowledge-map analysis. Front Oncol. (2021) 11:686726. 10.3389/fonc.2021.686726 34150654 PMC8209495

[22] Zhu X Hu J Deng S Tan Y Qiu C Zhang M. Bibliometric and visual analysis of research on the links between the gut microbiota and depression from 1999 to 2019. Front Psychiatry. (2020) 11:587670. 10.3389/fpsyt.2020.587670 33488420 PMC7819979

[23] Zhong W Shen Z Wu Y Mao X Kong J Wu W. Knowledge mapping and current trends of immunotherapy for prostate cancer: a bibliometric study. Front Immunol. (2022) 13:1014981. 10.3389/fimmu.2022.1014981 36389756 PMC9647028

[24] Liu K Zhao S Li J Zheng Y Wu H Kong J. Knowledge mapping and research hotspots of immunotherapy in renal cell carcinoma: a text-mining study from 2002 to 2021. Front Immunol. (2022) 13:969217. 10.3389/fimmu.2022.969217 35967367 PMC9367473

[25] Chen C. Searching for intellectual turning points: progressive knowledge domain visualization. Proc Natl Acad Sci U S A. (2004) 101:5303–10. 10.1073/pnas.0307513100 14724295 PMC387312

[26] Shen Z Hu J Wu H Chen Z Wu W Lin J. Global research trends and foci of artificial intelligence-based tumor pathology: a scientometric study. J Transl Med. (2022) 20:409. 10.1186/s12967-022-03615-0 36068536 PMC9450455

[27] Chen C. Science mapping: a systematic review of the literature. J Data Inform Sci. (2017) 2:1–40. 10.1515/jdis-2017-0006

[28] Chen C Hu Z Liu S Tseng H. Emerging trends in regenerative medicine: a scientometric analysis in citespace. Expert Opin Biol Ther. (2012) 12:593–608. 10.1517/14712598.2012.674507 22443895

[29] Li C Wu K Wu J. A bibliometric analysis of research on haze during 2000–2016. Environ Sci Pollut Res Int. (2017) 24:24733–42. 10.1007/s11356-017-0440-1 29034422

[30] van Eck NJ Waltman L. Software survey: vosviewer, a computer program for bibliometric mapping. Scientometrics. (2010) 84:523–38. 10.1007/s11192-009-0146-3 20585380 PMC2883932

[31] Xiang Y Zhai G Li Y Wang M Chen X Wang R. Ginkgolic acids inhibit SARS-CoV-2 and its variants by blocking the spike protein/ace2 interplay. Int J Biol Macromol. (2022) 226:780–92. 10.1016/j.ijbiomac.2022.12.057 36521705 PMC9743696

[32] Guo T Fan Y Chen M Wu X Zhang L He T. Cardiovascular implications of fatal outcomes of patients with coronavirus disease 2019 (COVID-19). JAMA Cardiol. (2020) 5:811–8. 10.1001/jamacardio.2020.1017 32219356 PMC7101506

[33] Wichmann D Sperhake JP Lütgehetmann M Steurer S Edler C Heinemann A. Autopsy findings and venous thromboembolism in patients with COVID-19: a prospective cohort study. Ann Intern Med. (2020) 173:268–77. 10.7326/L20-1206 32374815 PMC7240772

[34] Nobari NN Goodarzi A. Patients with specific skin disorders who are affected by COVID-19: what do experiences say about management strategies? A systematic review. Dermatol Ther. (2020) 33:e13867. 10.1111/dth.13867 32558193 PMC7323037

[35] Chen Q Wang L Li C Hu W Fan Y Chen Z. Chronic cardio-metabolic disease increases the risk of worse outcomes among hospitalized patients with COVID-19: a multicenter, retrospective, and real-world study. J Am Heart Assoc. (2021) 10:e018451. 10.1161/JAHA.120.018451 34096317 PMC8477891

[36] Manry J Bastard P Gervais A Le Voyer T Rosain J Philippot Q. The risk of COVID-19 death is much greater and age dependent with type I ifn autoantibodies. Proc Natl Acad Sci U S A. (2022) 119:e2200413119. 10.1073/pnas.2200413119 35576468 PMC9173764

[37] Raghavan S Gayathri R Kancharla S Kolli P Ranjitha J Shankar V. Cardiovascular impacts on COVID-19 infected patients. Front Cardiovasc Med. (2021) 8:670659. 10.3389/fcvm.2021.670659 34055939 PMC8155350

[38] Ellison-Hughes GM Colley L O'Brien KA Roberts KA Agbaedeng TA Ross MD. The role of msc therapy in attenuating the damaging effects of the cytokine storm induced by COVID-19 on the heart and cardiovascular system. Front Cardiovasc Med. (2020) 7:602183. 10.3389/fcvm.2020.602183 33363221 PMC7756089

[39] Breikaa RM Lilly B. The notch pathway: a link between COVID-19 pathophysiology and its cardiovascular complications. Front Cardiovasc Med. (2021) 8:681948. 10.3389/fcvm.2021.681948 34124207 PMC8187573

[40] Ercan H Schrottmaier WC Pirabe A Schmuckenschlager A Pereyra D Santol J. Platelet phenotype analysis of COVID-19 patients reveals progressive changes in the activation of integrin αiibβ3, f13a1, the SARS-CoV-2 target eif4a1 and annexin a5. Front Cardiovasc Med. (2021) 8:779073. 10.3389/fcvm.2021.779073 34859078 PMC8632253

[41] Khera R Clark C Lu Y Guo Y Ren S Truax B. Association of angiotensin-converting enzyme inhibitors and angiotensin receptor blockers with the risk of hospitalization and death in hypertensive patients with COVID-19. J Am Heart Assoc. (2021) 10:e018086. 10.1161/JAHA.120.018086 33624516 PMC8403305

[42] Pan M Vasbinder A Anderson E Catalan T Shadid HR Berlin H. Angiotensin-converting enzyme inhibitors, angiotensin ii receptor blockers, and outcomes in patients hospitalized for COVID-19. J Am Heart Assoc. (2021) 10:e023535. 10.1161/JAHA.121.023535 34889102 PMC9075226

[43] Matsushita K Marchandot B Jesel L Ohlmann P Morel O. Impact of COVID-19 on the cardiovascular system: a review. J Clin Med. (2020) 9:1407. 10.3390/jcm9051407 32397558 PMC7291320

[44] Bikdeli B Madhavan MV Jimenez D Chuich T Dreyfus I Driggin E. COVID-19 and thrombotic or thromboembolic disease: Implications for prevention, antithrombotic therapy, and follow-up: Jacc state-of-the-art review. J Am Coll Cardiol. (2020) 75:2950–73. 10.1016/j.jacc.2020.04.031 32311448 PMC7164881

[45] Chi W Chen Y Wang L Luo Z Zhang Y Zhu X. Acupuncture for COVID-19 patient after ventilator weaning: a protocol for systematic review and meta-analysis. Medicine. (2020) 99:e23602. 10.1097/MD.0000000000023602 33327327 PMC7738103

[46] Xiao F Sun R Sun W Xu D Lan L Li H. Radiomics analysis of chest ct to predict the overall survival for the severe patients of COVID-19 pneumonia. Phys Med Biol. (2021) 66:105008. 10.1088/1361-6560/abf717 33845467

[47] Xu D Zhou F Sun W Chen L Lan L Li H. Relationship between serum severe acute respiratory syndrome coronavirus 2 nucleic acid and organ damage in coronavirus 2019 patients: a cohort study. Clin Infect Dis. (2021) 73:68–75. 10.1093/cid/ciaa1085 32720678 PMC7454386

[48] Zhou F Yu T Du R Fan G Liu Y Liu Z. Clinical course and risk factors for mortality of adult inpatients with COVID-19 in wuhan, china: a retrospective cohort study. Lancet. (2020) 395:1054–62. 10.1016/S0140-6736(20)30566-3 32171076 PMC7270627

[49] Shi S Qin M Shen B Cai Y Liu T Yang F. Association of cardiac injury with mortality in hospitalized patients with COVID-19 in wuhan, china. JAMA Cardiol. (2020) 5:802–10. 10.1001/jamacardio.2020.0950 32211816 PMC7097841

[50] Guan WJ Ni ZY Hu Y Liang WH Ou CQ He JX. Clinical characteristics of coronavirus disease 2019 in china. N Engl J Med. (2020) 382:1708–20. 10.1056/NEJMoa2002032 32109013 PMC7092819

[51] Hoffmann M Kleine-Weber H Schroeder S Krüger N Herrler T Erichsen S. SARS-CoV-2 cell entry depends on ace2 and tmprss2 and is blocked by a clinically proven protease inhibitor. Cell. (2020) 181:271–280.e278. 10.1016/j.cell.2020.02.052 32142651 PMC7102627

[52] Huang C Wang Y Li X Ren L Zhao J Hu Y. Clinical features of patients infected with 2019 novel coronavirus in Wuhan, China. Lancet. (2020) 395:497–506. 10.1016/S0140-6736(20)30183-5 31986264 PMC7159299

[53] Salah HM Fudim M O'Neil ST Manna A Chute CG Caughey MC. Post-recovery COVID-19 and incident heart failure in the national COVID cohort collaborative (n3c) study. Nat Commun. (2022) 13:4117. 10.1038/s41467-022-31834-y 35840623 PMC9284961

[54] Tajbakhsh A Gheibi Hayat SM Taghizadeh H Akbari A Inabadi M Savardashtaki A. COVID-19 and cardiac injury: clinical manifestations, biomarkers, mechanisms, diagnosis, treatment, and follow up. Expert Rev Anti Infect Ther. (2021) 19:345–57. 10.1080/14787210.2020.1822737 32921216

[55] Mizera L Borst O. COVID-19 and the incidence of acute myocardial injury. Hamostaseologie. (2021) 41:356–64. 10.1055/a-1554-6416 34695852

[56] Giustino G Croft LB Stefanini GG Bragato R Silbiger JJ Vicenzi M. Characterization of myocardial injury in patients with COVID-19. J Am Coll Cardiol. (2020) 76:2043–55. 10.1016/j.jacc.2020.08.069 33121710 PMC7588179

[57] Hu Y Liu K Han P Xu Z Zheng A Pan X. Host range and structural analysis of bat-origin rshstt182/200 coronavirus binding to human ace2 and its animal orthologs. EMBO J. (2022) 2022:e111737. 10.15252/embj.2022111737 36519268 PMC9877840

[58] Oh CK Nakamura T Beutler N Zhang X Piña-Crespo J Talantova M. Targeted protein s-nitrosylation of ace2 inhibits SARS-CoV-2 infection. Nat Chem Biol. (2022) 2022:1–9. 10.1038/s41589-022-01149-6 36175661 PMC10127945

[59] Torchia JA Tavares AH Carstensen LS Chen DY Huang J Xiao T. Optimized ace2 decoys neutralize antibody-resistant SARS-CoV-2 variants through functional receptor mimicry and treat infection *in vivo*. Sci Adv. (2022) 8:eabq6527 10.1126/sciadv.abq6527 36475798 PMC9728973

